# Shikonin induces colorectal carcinoma cells apoptosis and autophagy by targeting galectin-1/JNK signaling axis

**DOI:** 10.7150/ijbs.36955

**Published:** 2020-01-01

**Authors:** Nan Zhang, Fu Peng, Yujia Wang, Li Yang, Fengbo Wu, Xiaoyun Wang, Cui Ye, Bo Han, Gu He

**Affiliations:** 1 State Key Laboratory of Southwestern Chinese Medicine Resources, School of Pharmacy, Chengdu University of Traditional Chinese Medicine, Chengdu 611137, China; 2 State Key Laboratory of Biotherapy and Cancer Center, West China Hospital, and West China School of Pharmacy, Sichuan University, Chengdu 610041, China

**Keywords:** Shikonin, Colorectal carcinoma, Galectin-1, apoptosis, autophagy

## Abstract

Colorectal carcinoma (CRC) is the third most common malignant tumor pathology worldwide. Despite progress in surgical procedures and therapy options, CRC is still a considerable cause of cancer-related mortality. In this study, we tested the antitumor effects of shikonin in CRC and tried to identify its potential mechanism. The potential target, molecular mechanism as well as *in vitro* and *in vivo* antitumor effects of shikonin in CRC cells were determined by an integrative protocol including quantitative proteomics, RT-PCR, western blotting, RNA interference and overexpression, apoptosis and autophagy assays, etc. Galectin-1 was a potential target of shikonin from the iTRAQ-based proteomic analysis in shikonin-treated SW620 cell. The overexpression and RNA silencing of galectin-1 in two CRC cells suggested that the shikonin sensitivity was correlation to galectin-1 levels. The ROS accumulation induced by shikonin was important to the formation of galectin-1 dimers. Dimer galectin-1 was found to be associated with the activation of JNK and downstream apoptosis or autophagy. Moreover, through functional *in vitro* studies, we showed that differences in galectin-1 level affected tumor cell proliferation, migration, and invasion. In summary, shikonin induced CRC cells apoptosis and autophagy by targeting galectin-1 and JNK signaling pathway both *in vitro* and *in vivo*, which suggested a potential novel therapy target for CRC.

## Introduction

Colorectal cancer is a disease caused by malignant cells growth in the tissues of the colon or rectum. It is the third most common cancer behind lung and breast cancer worldwide, and the mortality rate is second only to malignant tumors of lung cancer.^1^ Early screening has been shown to reduce the incidence and mortality of colorectal cancer.^2^ At present, the treatment of primary and metastatic colorectal cancer mainly includes laparoscopic surgery for primary diseases, resection of metastatic lesions, adjuvant radiotherapy (for patients with rectal cancer) and adjuvant chemotherapy (for patients with stage III/IV and high-risk stage II colon cancer). The five-year survival rate ranged from 90% of patients with stage I disease to more than 10% of patients with stage IV disease.^3^, ^4^ Despite the tremendous progress in surgical and medical treatments, the impact of these treatments on the cure rate and long-term survival rate of colorectal cancer is limited, and more effective treatments are urgently needed.

In recent years, research and development of natural product based antitumor agents has made great progress. Many chemotherapeutics for clinical application are synthetic natural product derivatives or extracted from plant sources.^5^-^11^ One-third of the chemical antitumor drugs are derived from plant sources.^12^-^14^ Shikonin and its derivatives, main effective components of *Lithospermum erythrorhizon*, have been proven to have many kinds of biological activities.^15^-^17^ The study of the antitumor effects of shikonin has made some progress with the discovery of effective derivatives that have larger research value.^18^-^20^ However their specific molecular mechanisms are still unclear. The detailed antitumor mechanism involving multiple targets remained to be further confirmed.

Galectins are a family of proteins thatcan specifically binds to β-galactosides. Galectins have a broad variety of biological functionsand influence multiple diseases.^21^-^23^ Galectin-1 (Gal-1) has multiple functions both intracellular and extracellular being expressed within and outside of cells. The carbohydrate-binding properties of dGal-1 were shown to be necessary to these extracellular functions.^24^-^27^ On the contrary, the interactions between Gal-1 and other proteins such as α5β1 integrin, which can interact with Gal-1 and inhibit cell growth, were independent carbohydrates for intracellular parts.^28^-^30^ The classical signal pathway Ras-MEK-ERK also has been found to participate in Gal-1-related antiproliferative effects.

Current studies have shown that shikonin can inhibit the pro-inflammatory ability produced during the onset of acute colitis, inhibits the proliferation of colorectal cancer cells, and promotes apoptosis of colorectal cancer cells. Some research also showed that shikonin can be used as a chemotherapeutic drug cisplatin by activating intracellular oxidative stress.^31^-^33^ In the our study, we examined the antitumor effects of shikonin in CRC *in vitro* and *in vivo* and elucidated that shikonin induced the production of ROS and dimeration of galectin-1, which was found associated with the sensitivity of CRC cell lines to shikonin. Furthermore, we investigated that shikonin administration inhibited tumor growth on tumor xenograft model. These results suggest that shikonin is a promising antitumor agent, and can play an anti-colorectal cancer role by modulating the galectin-1/JNK signaling pathway.

## Materials and methods

### Cell lines and Animals

SW620 cell line and HCT116 cell line (human colorectal adenocarcinoma) were obtained from the Type Culture Collection of the Chinese Academy of Sciences, Shanghai, China. SW620 cell was grown in DMEM (Hyclone, USA) supplemented with 10% fetal bovine serum (FBS, Gibco, USA). All the cells were maintained at 37°C in a humidified incubator containing 5% CO_2_. Balb/c nude mice (6-8 weeks) purchased from Vital River (Beijing, China) were used for the *in vivo* experiments. We provided all the animals a house with controlled temperature of 20-22°C, relative humidity of 50-60%, and 12h light-dark cycles. All animal expriments were performed based on the protocol approved by the Institutional Animal Care and Treatment Committee of Sichuan University (Chengdu, PR China).

### Chemicals and Antibodies

Shikonin was obtained from Selleckchem Co. Ltd. (Shanghai, China). The stock solution of 40 mM was prepared by dissolving in DMSO. DCFH-DA was from Sigma-Aldrich (Munich, Germany); SP600125 was from Alexis Biochemicals (San Diego, CA, USA); Rapamycin, 3-MA and Bafilomycin A1 were from Selleck; HCQ and N-acetyl-L-cysteine (NAC) were from Sigma (St. Louis, MO, USA). The antibodies used were as following: JNK, phospho-JNK, Bcl-2, Bax, caspase 8, ATG5, LC3, p62 and Beclin-1, which were from Cell Signaling Technology; caspase 3, caspase 9, PARP, Fas, Fasl, Galectin-1, and Ki67, which came from Abcam (Chicago, IL, USA); Glyceraldehyde-3-phosphate dehydrogenase (GAPDH), β-actin and horseradish peroxidase-conjugated affinipure goat anti-mouse and anti-rabbit IgG, which came from ZSGB-BIO (Beijing, China).

### Cell viability and Colony Formation Assays

Cell viability was determined by MTT (Sigma-Aldrich) assays according to established protocols. SW620 cell seeded in 96-well plates were treated by a series of concentration shikonin for 24h. The mean percentage of cell survival rates was determined from data of three individual experiments. Cells were seeded in six-well plates at 8 × 10^2^ cells per well following by treating with different concentration of shikonin. After incubation for enough time (almost 2 weeks) for the colony formation assay, the cells were then washed twice with cold PBS, fixed with 4% paraformaldehyde, and stained with 0.5% crystal violet (Sigma, St Louis, MO, USA).

### Apoptosis and Autophagy assays

For apoptosis assays, SW620 cell cultured in 6-well plates for 24h were exposed to media containing 0,3,6,12μM shikonin for another 24h. Then fix the cells with 4% paraformaldehyde for 10min and stain with 0.2ml Hoechst33258 (1 μg/ml in H_2_O) for 10min. The nuclear shrinkage and chromatin condensation were found in apoptptic cells by fluorescence microscopy (Olymbus). For further step, flow cytometric (FCM) analysis was performed to confirm the apoptotic induction abilities of shikonin. Cells treated by shikonin as before were harvested and washed with PBS, resuspended in binding buffer from Roche, stained with Annexin V-FITC and propidium iodide (PI) for 15min. The early or late apoptotic cells were identified by flow cytometry (BD Biosciences, USA). GFP-LC3-transfected SW620 and HCT116 cells were utilized to performing the autophagy assay. The GFP-LC3-transfected cells were treated with shikonin for 36h. The aggregation of GFP-LC3 in the two colorectal carcinoma cell lines was observed by a fluorescence microscope, which means the occurrence of autophagy.

### Detection of ROS

To investigate the effect of shikonin on ROS, SW620 cell were treated with 0,3,6,12μM shikonin for 24h. Then, cells were collected and incubated with 10 μM 2'-7'-dichlorouorescein diacetate (DCFH-DA) at 37°C for 25 min. The ROS-induced fluorescence intensity of intracellular DCFH-DA was measured by FCM.

### Cell transfection and RNA interference by siRNA

The galectin-1 over-expression (OE-Gal1) plasmid pCDH-Gal1-puro was synthesized (GENEWIZ, Suzhou, China). The lipofectamine 2000 reagent (Life Technologies Corporation, Carlsbad, CA, USA) was used for the cells transfection according to the manufacturer's instructions. 293T cells were cultured in 100-mm dishes at 37°C 5% CO2. 10μg pCDH-LGALS1-puro and 7μg pXPAX, 3 μg pMD2.G were transfected into 293T cells, when grown to about 60% confluence to made lentiviral OE-Gal1. Empty vector was used as a control. Subsequently, when grown to about 70-80% confluence, SW620 and HCT116 cells were infected with the above lentiviral OE-Gal1 in the presence of 5 mg/ml polybrene, and exposed to 4 μg/ml puromycin (Sigma-Aldrich, St Louis, MO, USA) after 24 h of infection. After a 5-day puromycin management, cells were used for experiments. The SW620 cell lines and HCT116 cell lines were transfected with siRNA by Lipofectamine 2000 (Invitrogen, USA) according to the manufacture's protocol. The sequence of galectin-1 siRNA as below: Si-gal1 1#: GCTGCCAGATGGATACGAA; and Si- gal1 2#: TGCCAGATGGATACGAATT; Si-gal1 3#: GGCCATCAACTACATGGCA; the negative control siRNA (si-NC) were purchased from Ribobio, Guangzhou, China.

### DARTS assay

Drug affinity responsive target stability (DARTS) assay were performed following a previously described method. Cells were lysed with M-PER (Pierce) supplemented with protease and phosphatase inhibitors for 10min and centrifuged at 14,000 rpm for 15min at 4°C. Then lysates were diluted to the same final volume and protein concentration with M-PER and proteolysed in reaction buffer [50 mM Tris-HCl (pH 8.0), 50 mM NaCl, 10 mM CaCl_2_]. All steps were performed on ice to prevent premature protein degradation. Lysates were incubated with DMSO or different concentration of shikonin at room temperature for 4h and then proteolysed with 1μg of pronase for every 300μg of lysate for 30min. To stop proteolysis, 1×SDS loading buffer was added and heated as above immediately. Samples were loaded on SDS-PAGE. At the end, gels were stained with Coomassie or perform Western-blot analysis. 40 ng/μL recombinant galectin-1 was incubated with shikonin or DMSO solvent control overnight at 4°C, followed by digestion with Pronase 1:100 (wt:wt) at room temperature. Then added 1 × SDS loading buffer to stop proteolysis, and heating as above immediately. Samples were loaded on SDS-PAGE. At the end, gels were stained with Coomassie or perform Western-blot analysis.

### Subcutaneous tumor mouse model

To investigate the anti-tumor activity of shikonin, a subcutaneous SW620 tumor mouse model was used. 100μL of SW620 cell suspension (1×10^7^ cells) were injected subcutaneously into the right dorsal flank of mice at day 0. When the tumors were tangible, mice were randomized into two groups (18 mice per group). Since day 4, mice were injected intraperitoneally with 100 μL of normal saline (NS, control), or shikonin (1.5 mg/kg) every 24h. The tumor size was measured every third day using digital calipers. Mice in the control group began to die at day 20, and all animals were scarified with tumor removed. Tumor tissue were fixed in 4% paraformaldehyde and stained with antibodies for ATG5, Beclin-1, LC3, galectin-1 and Ki67. Tumor tissue were fixed in 4% paraformaldehyde, embedded in paraffin, and sectioned into 4μm slices. Terminal deoxynucleotidyl transferase-mediated nick-end labeling (TUNEL) staining was preformed according to the manufacturer's protocol for TUNEL assay kit (Promega, Amercia) and five equal-sized sections were chosen randomly and calculated the ratio of the apoptotic cell.

## Results

### Shikonin inhibits cell proliferation and induces apoptosis of colorectal cancer cells

**Figure 1:**
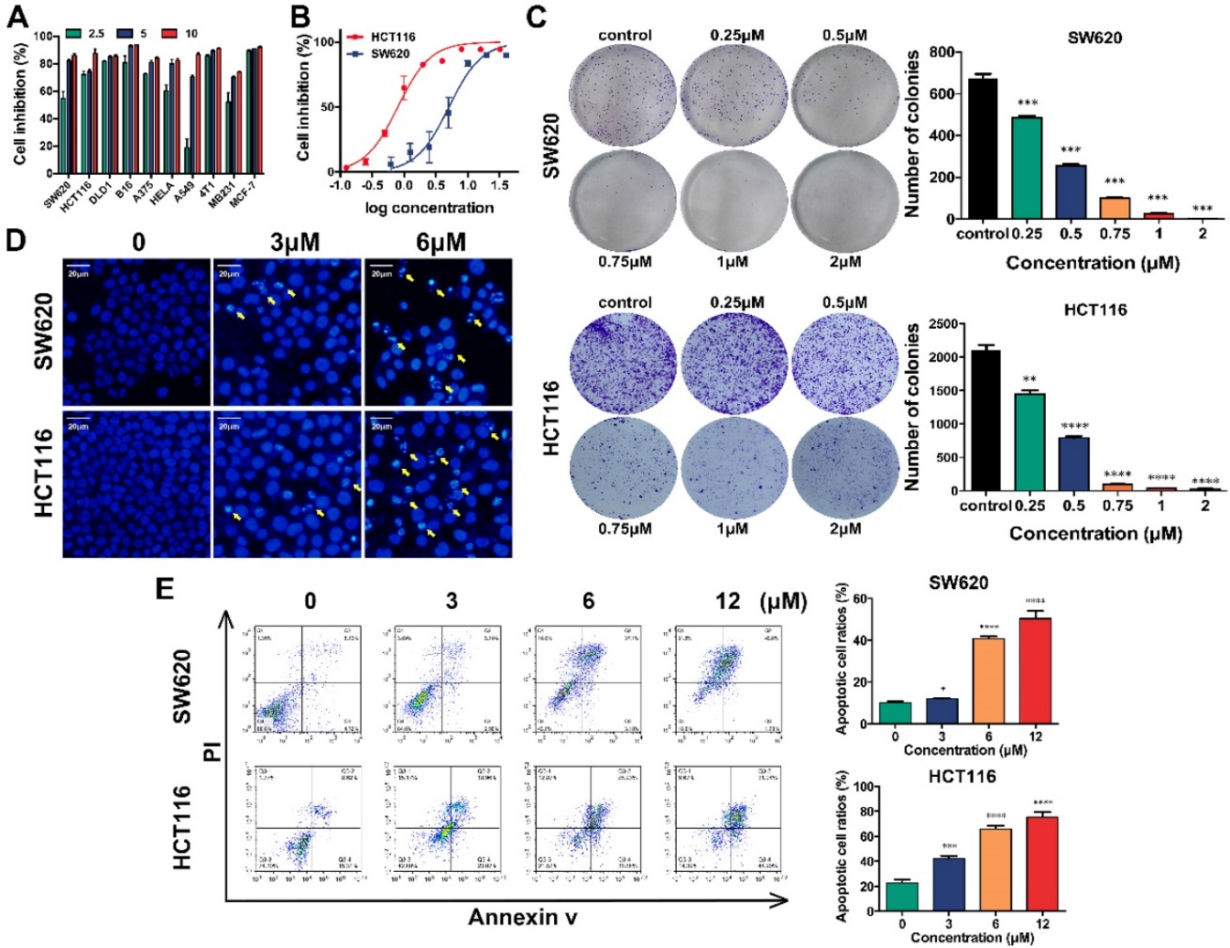
(A) The cell proliferation inhibition (%) of a panel of cancer cell lines incubated by shikonin for 24h; The green color label for 2.5μM shikonin-treated group, while the blue color label for 5μM shikonin-treated group and red color label for 10μM; (B) The cell proliferation inhibition curves of SW620 and HCT116 colorectal carcinoma cells after shikonin incubation; (C) The colony formation of SW620 and HCT116 cells after shikonin incubation; (D) The nuclei morphological changes of SW620 and HCT116 cells incubated by shikonin with Hoechst33258 staining; (E) The apoptosis assays of SW620 and HCT116 cells incubated by shikonin. (*, p < 0.05, ****, p < 0.0001)

**Figure 2:**
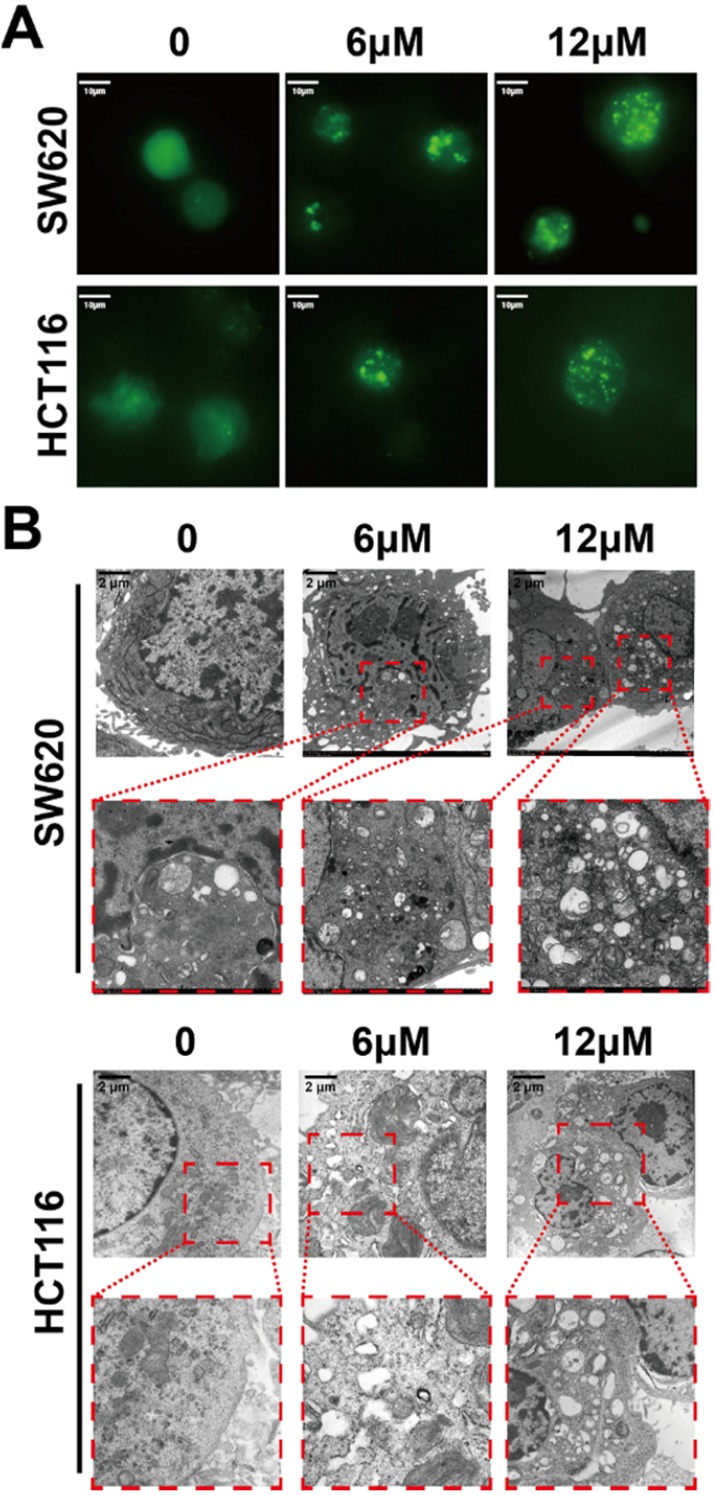
(A) The formation of GFP-LC3 fluorescent puncta induced by shikonin incubation; (B) The autophagic vacuoles induced by shikonin incubation in SW620 and HCT116 cells detected by TEM.

The *in vitro* cytotoxicity of shikonin for different kinds of tumor cells was determined using cell viability assays of ten cell lines. All of the tested cell lines were treated with 2.5μM, 5μM and 10μM shikonin for 24h and had increasingly poor cell viability with increases in drug concentration (Figure 1A). The IC_50_ of shikonin in SW620 and HCT116 cells was 3-6 μM (Figure 1B). The colony formation abilities of SW620 and HCT116 cells were inhibited after shikonin incubation in a dose dependent manner (Figure 1C and Figure S1). Moreover, the apoptotic nuclei condensation induced by shikonin were observed by fluorescence microscopy in both SW620 and HCT116 cells after Hoechst33258 staining (Figure 1D). Then different administration of shikonin were evaluated by flow cytometry on SW620 and HCT116 cells undergoing early as well as late apoptosis and were differentiated based on the ability of Annexin V-FITC/PI to stain phosphatidylserine on the outer membrane and DNA fragmentation of the apoptotic cells.^34^-^36^ Treatments with higher concentrations resulted in an increased percentage of apoptotic cells compared with control group (Figure 1E). The initiation of autophagy was detected by GFP-LC3 fluorescent puncta and autophagic vacuole in fluorescent microscopy and transmission electronic microscopy, respectively (Figure 2).

### Potential signaling pathways and targets for shikonin-induced cell programmed death assessed by quantitative proteomic analysis (iTRAQ)

**Figure 3:**
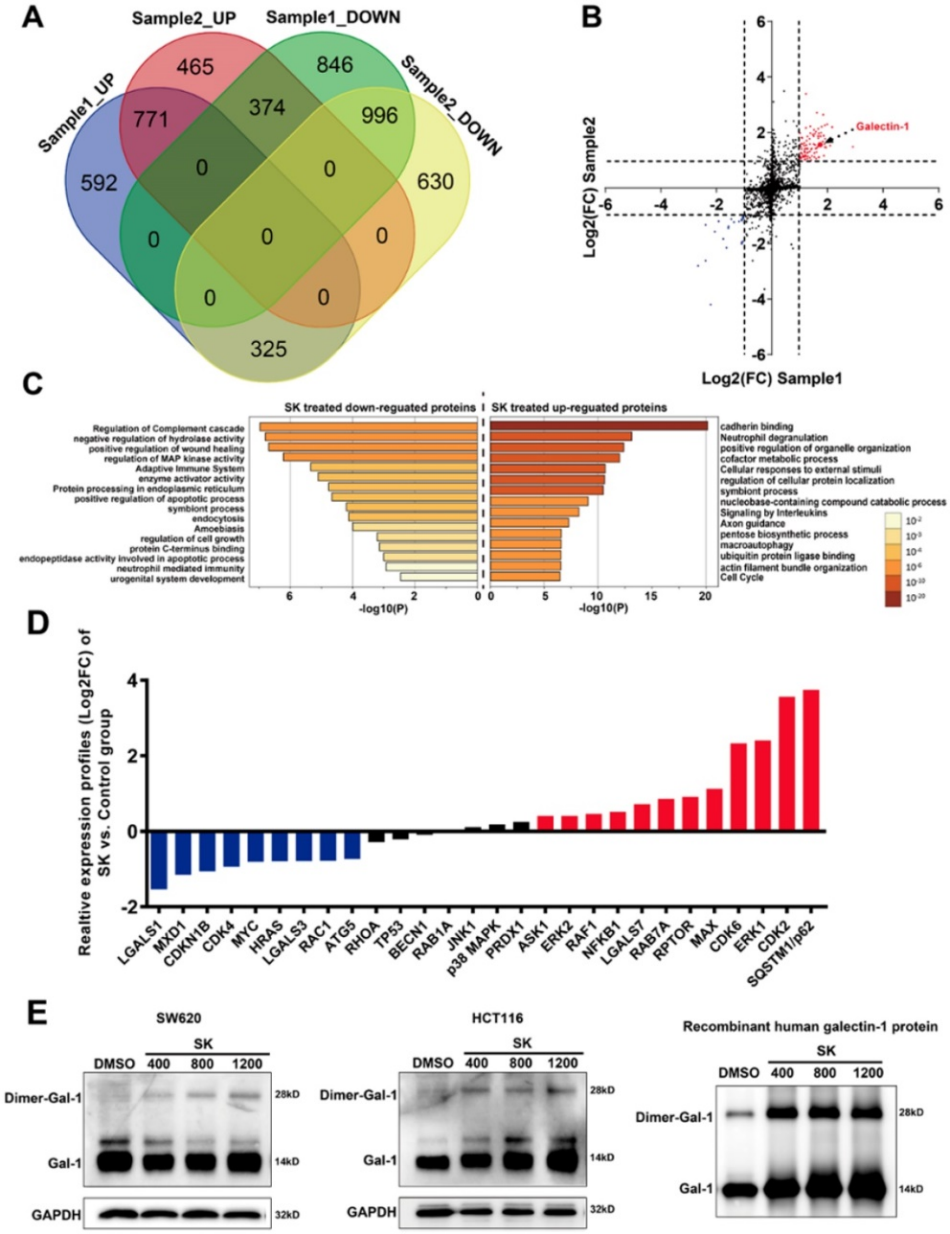
iTRAQ-based analysis of shikonin-related signaling pathways. (A) (B) (C) Cluster enrichment of the differentially expressed proteins in shikonin-treated SW620 cell; (D) Real-time PCR analysis of different levels of mRNA in shikonin (SK)-treated SW620 cell; (E) Target identification for interaction between shikonin (SK) and galectin-1 by DARTs. Cell lysates and recombinant galectin-1 were both incubated with 400μM, 800μM and 1200μM shikonin or DMSO.

To further determine the potential mechanisms related to shikonin-induced programmed cell death, a quantitative proteomic analysis based on iTRAQ (isobaric tag for relative and absolute quantitation) methodology was utilized to identify differentially expressed proteins in shikonin-treated SW620 cell. There were over 5,000 proteins detected in the iTRAQ results with 95% confidence; only 123 proteins were significantly differentially expressed (|Log_2_FC| >1) between control and shikonin-treated SW620 cell (99 up-regulated proteins and 24 down-regulated proteins, see Figure 3A and Table S1). Pathway enrichment analysis results demonstrated that cadherin binding, macro-autophagy, regulation of apoptosis and the MAPK pathway were significantly enriched in differentially expressed proteins (Figure 3C).

Moreover, the analysis of PPI (protein-protein interaction) networks indicated that the interaction network between HRAS and galetin-1 was an important intermediator of this PPI network (Figure 3B). Then we identified changes to the expression of some important intermediator of this PPI network at mRNA levels via qPCR. Our results demonstrated many genes related to apoptosis, autophagy and MAPK pathways were significantly upregulated, including Galectin -7, Raf1, Myc, ATG5, SQSTM1, JNK1, ERK1/2, p38MAPK, etc (Figure 3D). In order to identify proteins that may possibly interact with shikonin, we carried out DARTs against SW620 and HCT116 cell protein extracts. DARTs assay is a strategy based on the reduction of the protease susceptibility of the target protein when binding with drug. Interestingly, both galectin-1 and galectin-1 dimer in cell lysate was identified as interacting with shikonin in both cell lines. Furthermore, we performed proteolysis of recombinant human galectin-1 with the protease pronase. After incubated with 400μM, 800μM and 1200μM shikonin, the degradation of galectin-1 decreased, which suggested shikonin displayed significant protection for galectin-1 in resisting degradation in a dose-dependent manner (Figure 3E).

### Shikonin induces apoptosis and inhibits autophagic flux of colorectal cancer cells

**Figure 4:**
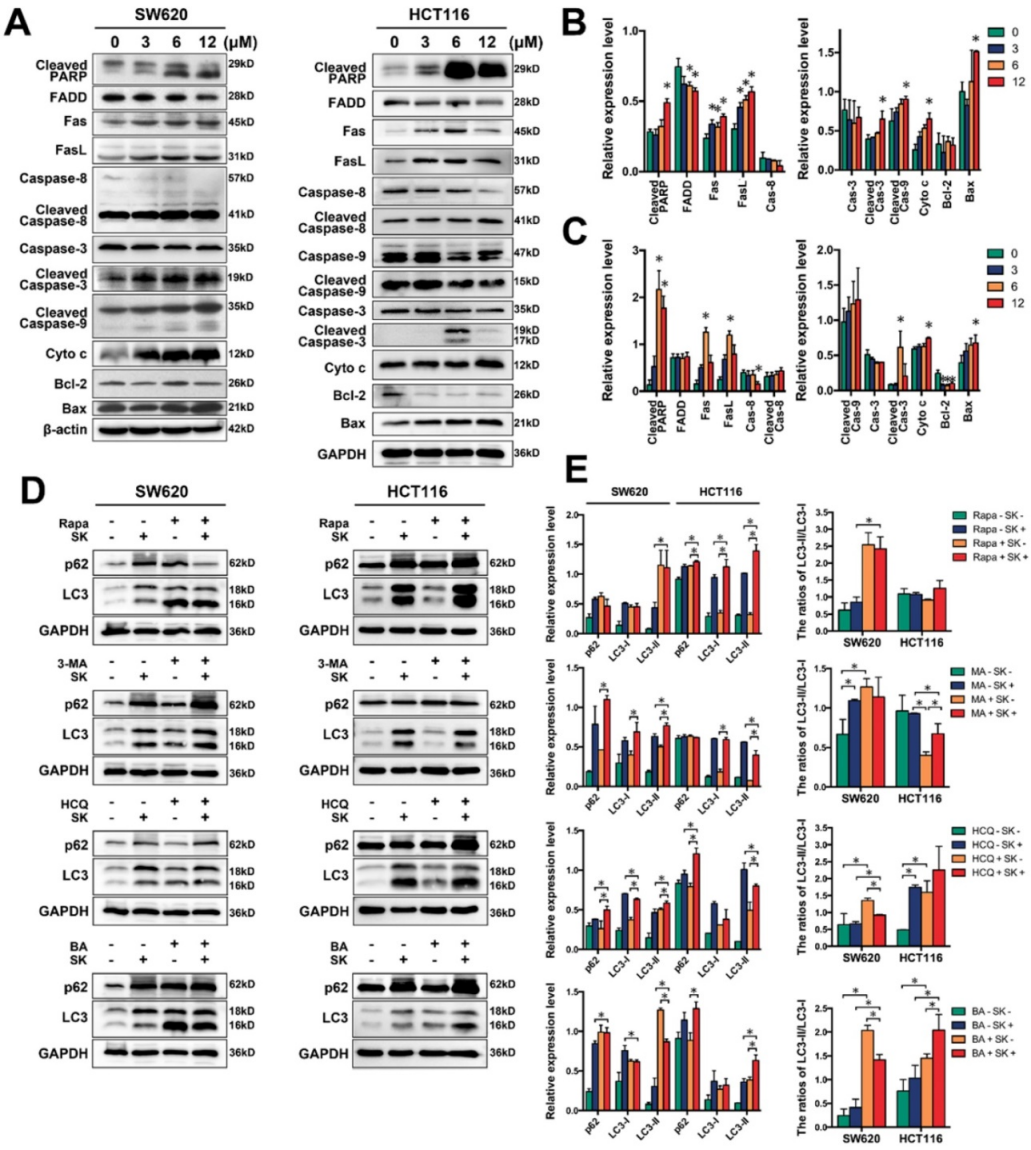
(A) Western bloting of apoptotic markers; (B-C) Quantification of apoptotic markers in panel A for SW620 and HCT116 cells; (D) Western bloting of p62 and LC3 in total lysates of SW620 and HCT116 cells after treated with shikonin (SK) alone or the combination of shikonin (SK) and autophage activator or inhibitor; (E) Quantification of autophagic markers in panel D. “Rapa” represents the autophage activator rapamycin. “3-MA” represents the autophage inhibitor 3-methyladenine. “HCQ” represents hydroxychloroquine and “BA” represents bafliomycin and both of HCQ and BA are autophage inhibitors can inhibit the degradation of autophagolysosomes.

We examined classical apoptosis-related proteins by western blot and found caspase-3 and PARP cleaved after treated with shikonin. We also found the proteins of mitochondrial apoptosis, such as Bax, cytochrome c were upregulated and the cleavage of caspase-9 were increase. The proteins of death receptor apoptosis, such as Fas, caspase-8, clearly experienced changes. All these results confirmed shikonin induced cell apoptosis via activating both of the two pathways (Figure 4A-C).

Shikonin also significantly induced the formation of autophagosome in both two CRC cell lines by promoting LC3 cleavage. But shikonin induced p62 accumulation that indicated shikonin might inhibit autophagic flux by blocking the degradation of autophagolysosome. Treating with the autophagy activator rapamycin (100nM, 3h) could drastically improving the cleavage of LC3 and promoting the shikonin-induced cell death (Figure 4D and S1).^37^ Shikonin-treatment along with the known autophage inhibitor 3-MA (10μM), Bafilomycin A1 (100nM) and HCQ (50μM) 3h early than shikonin enhanced shikonin-induced accumulation of p62 and LC3. All these results suggest that shikonin might be able to induce autophagic death by induced autophagy but inhibit the degradation of autophagolysosome.

### Shikonin activates apoptosis and autophagy by upregulating levels of ROS in colorectal cancer cells

**Figure 5:**
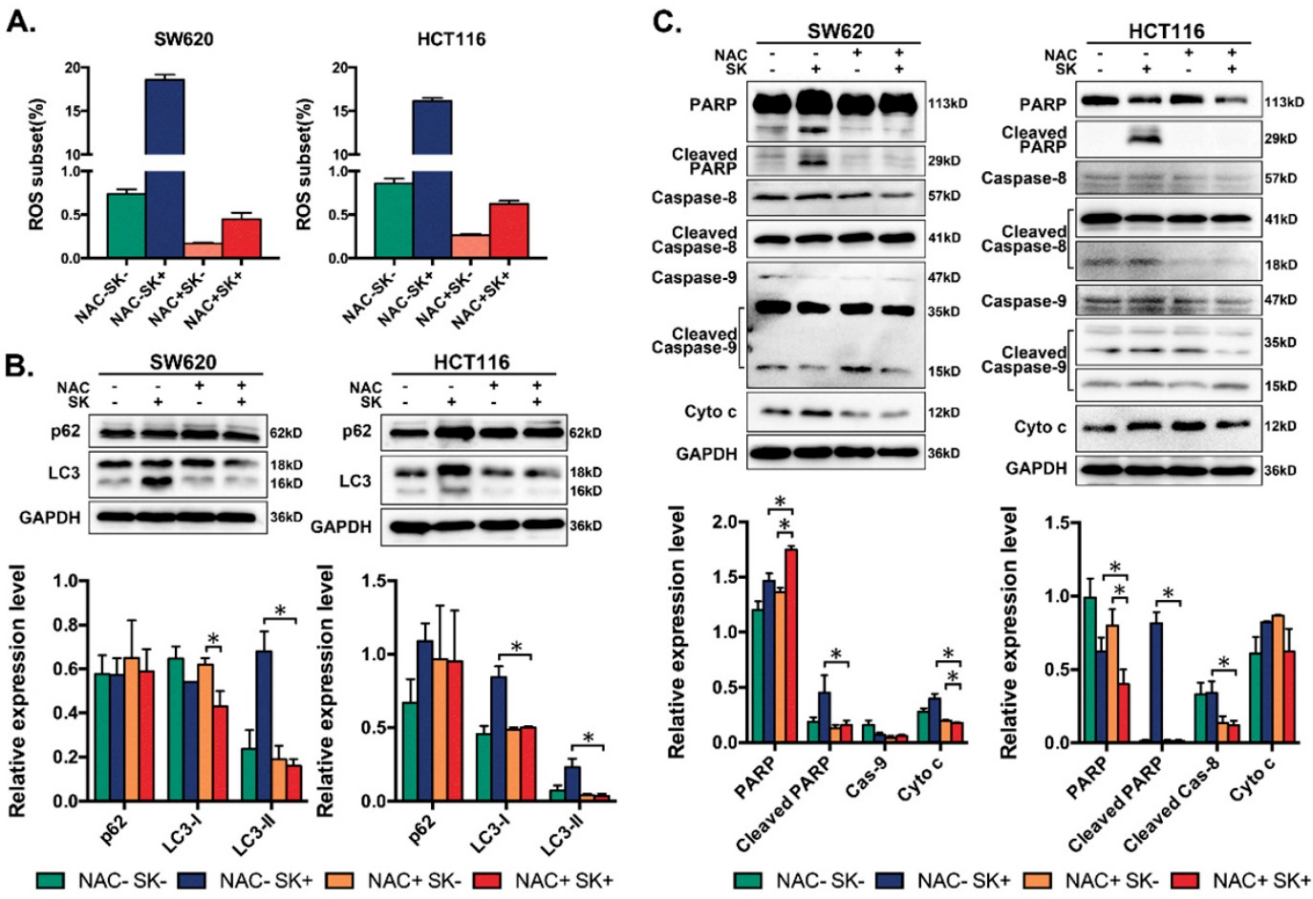
(A) Flow cytometry based on DCFH-DA staining to assess levels of ROS in two CRC cell lines after treated with shikonin (SK) alone or the conmbination of shikonin (SK) and NAC; (B) Western bloting of p62 and LC3 in total lysates of SW620 and HCT116 cells after treated with shikonin (SK) alone or the combination of shikonin (SK) and NAC; (C) Western bloting of apoptotic markers in total lysates of SW620 and HCT116 cells after treated with shikonin (SK) alone or the combination of shikonin (SK) and NAC.

Many types of chemotherapeutic drugs can activate the accumulation of reactive oxygen species (ROS) in cells, which can induce apoptosis or autophagy.^38^-^41^ Shikonin-induced apoptosis has been reported associated with ROS in chronic myelogenous leukemia cells.^42^ In further studies we also found shikonin could resulted the accumulation of ROS in CRC cells, which could induce apoptosis directly (Figure 5A and Figure S2). When treated with 5mM NAC to remove ROS, classical apoptosis-related proteins change their expression, cleaved PARP decreased and shikonin fail to upregulate the expression of cytochrome c visibly (Figure 5C). In addition, downregulation of ROS also decreased the cleavage of LC3, which means ROS plays an important role in shikonin-induced autophagy (Figure 5B).

### Galectin-1 plays an important role in shikonin-induced programmed cell death

**Figure 6:**
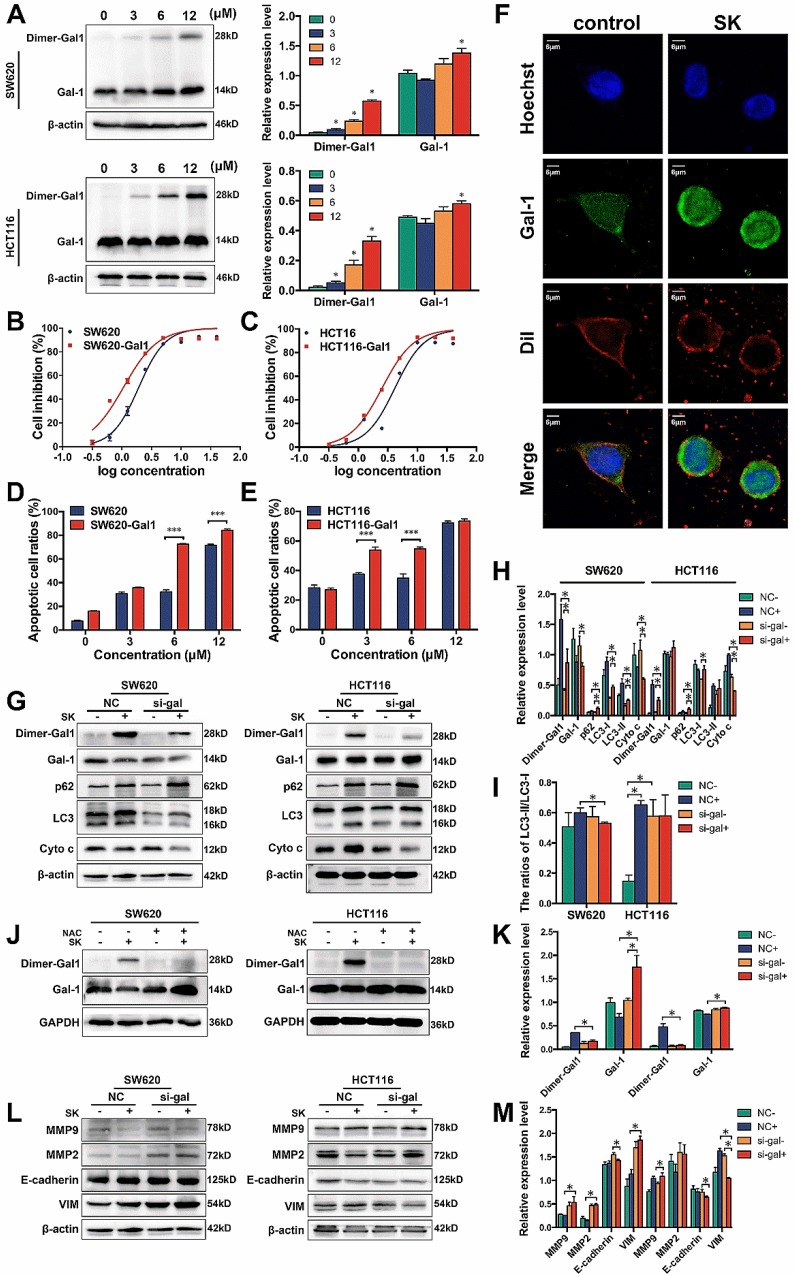
(A) Western bloting of galectin-1 in total lysates of SW620 and HCT116 cells after treated with shikonin; (B) Cell inhibition ratios in SW620 and galectin-1 overexpressed SW620 (SW620-Gal1); (C) Cell inhibition ratios in HCT116 and galectin-1 overexpressed HCT116 (SW620-Gal1); (D) Quantification of flow cytometry analysis to assess total apoptotic ratios in SW620 and SW620-Gal1 after treated with shikonin; (E) Quantification of flow cytometry analysis to assess total apoptotic ratios in HCT116 and HCT116-Gal1 after treated with shikonin; (F) Laser confocal microscopy images; (G-I) Changes in expression of Dimer-gal1, gal-1, p62, LC3 and Cyto c after treatment with si-gal1, shikonin (SK) or both; (J-K) Changes in expression of Dimer-gal1 and gal-1 after treatment with shikonin (SK), NAC or both; (L-M) Changes in expression of EMT markers after treatment with si-gal1, shikonin (SK) or both.

Galectin family is one class of β-galactoside-binding lectins that possesses diverse extracellular and intracellular effects. Galectins have been reported to play an important role in several cellular process, including apoptosis, cell adhesion, and the immune response.^43^-^45^ In the current manuscript, we have revealed that galectin-1 may interact with shikonin and be highly related to shikonin-induced programmed cell death. Our results also indicated that shikonin could up-regulate the expression and promote the dimerization of galectin-1 (Figure 6A&F). In order to further determine the role of galectins, especially galectin-1 in shikonin-induced colorectal carcinoma cells apoptosis, we overexpressed galectin-1 in SW620 and HCT116 cells and found the two cell lines became more sensitive to shikonin (Figure 6B-C). The overexpression group had a significantly greater increase in apoptotic cells compared with the control group when treated with the same concentration of shikonin (Figure 6D-E).

Furthermore, we downregulated the level of galectin-1 in colorectal carcinoma cells by siRNA and evaluated whether this process would influence cell apoptosis. Galectin-1 was decreased and some apoptosis-related proteins, such as cytochrome c were reduced after treatment with shikonin (Figure 6G-I). This change meant galectin-1 plays an essential role in shikonin-induced apoptosis just like ROS. We also found the increase of p62 by shikonin in both of the two cell lines were much higher when interfered the expression of galectin-1 and the shikonin induced cleavage of LC3 was decrease, which means the downregulation of galectin-1 and the dimer may hinder the autophage progress of CRC cell lines and promote the accumulation of p62. Then we investigated the relationship between galectin-1 and ROS, we added the ROS scavenger NAC, and the dimer of galectin-1, which plays the main role in shikonin-induced apoptosis, all disappeared (Figure 6J-K).

The ability of tumor cells to migrate from their primary site is critical to cancer progression and metastasis. SW620 and HCT116 cells have each displayed significant migratory and invasion potential previously. We tested the effects of shikonin on migration and invasion in SW620 and HCT116. Matrix metalloproteinases (MMPs) are proteolytic enzymes that can cleave or degrade almost all kinds of proteins in the ECM and destroy histologic barriers for tumor invasion and metastasis, enhance migration and invasion of tumor cells. We measured the expression of MMP2 and MMP9 with western blot and found shikonin could downregulate MMP2 but when decreases in Galectin-1 though shikonin did not (Figure 6L-M). These results confirmed shikonin could inhibit colorectal cancer cells invasion and Galectin-1 play an important role.

### Galectin-1 in shikonin-induced programed cell death via MAPK pathway

**Figure 7:**
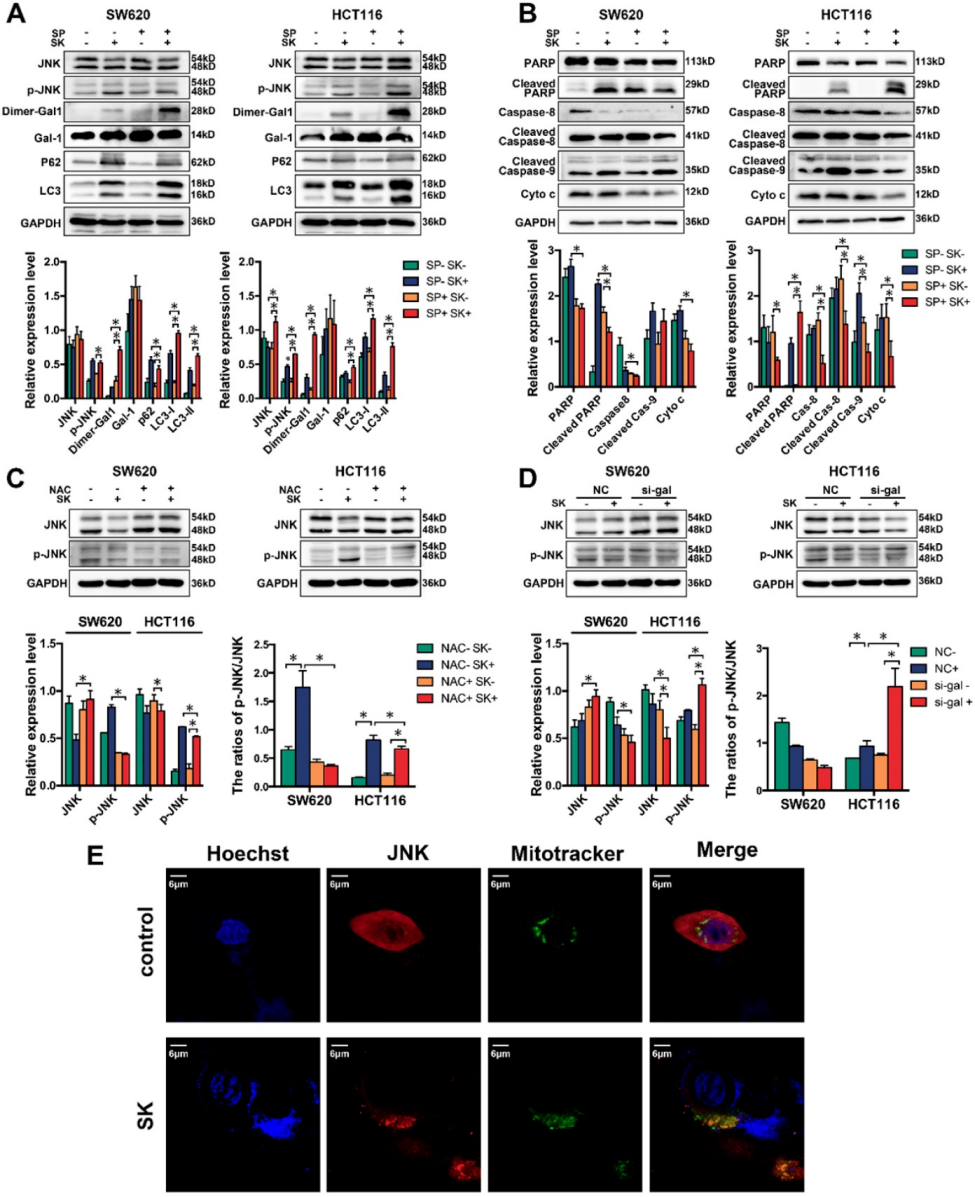
(A) Western bloting of Dimer-gal1, gal-1, LC3 and p62 after treatment with shikonin (SK) and JNK inhibitor (SP); (B) Western bloting of apoptotic markers after treatment with shikonin (SK) and JNK inhibitor (SP); (C) Western bloting of JNK and p-JNK after treatment with shikonin (SK) and NAC; (D) Western bloting of JNK and p-JNK after treatment with shikonin (SK) and siRNA for galectin-1 (si-gal); (E) Laser confocal microscopy images of cell location of JNK and mitochondria.

Galectin-1 interacts with Ras protein in the plasma membrane and cytoplasm, and then affects several cellular processes via the MAPK pathway.^46^-^48^ In order to identify whether MAPKs, such as JNK, could contribute to galectin-1-induced apoptosis, we employed the chemical inhibitor of JNK and treated SW620 and HCT116 cells with SP600125 (a JNK inhibitor) in the concentration of 20μM 3h earlier before shikonin. Western blot analysis showed that the protein levels of JNK were curbed by SP600125. Our results indicated the SP600125 upregulated the expression of dimer galectin-1 and enhanced shikonin-induced cell death (Figure 7A-B). Colorectal cancer cells (SW620 and HCT116) treated with SP600125 and shikonin at the same time promoted the degradation of caspase and PARP when compared with shikonin treatment alone (Figure 7B). The same phenomenon was observed for LC3, a major biomarker of autophagy. In addition, we detected the expression and activation of JNK in two CRC cell lines treated with NAC, and found that the activation level of JNK by shikonin in SW620 cell was significantly reduced, which indicating that ROS induced by shikonin had an important effect on the activation of JNK (Figure 7C). We also detected the expression and activation of JNK in cells transfected the siRNA of Galectin-1. In HCT116 cell, the down-regulation of Galectin-1 expression could significantly increase the phosphorylation level of JNK, suggesting that Galectin-1 may have a negative regulatory effect on JNK activation (Figure 7D).

Shikonin can induce mitochondrial-dependent cell apoptosis, which mainly affects mitochondrial permeability by regulating the expression of Bcl-2 family proteins and triggers the release of cytochrome c and other pro-apoptotic factors from mitochondria. JNK plays an important role in mitochondrial pathway of apoptosis. JNK phosphorylates Bcl-2 family proteins and promotes Bax to translocate from cytoplasm to mitochondria, resulting in changes in mitochondrial membrane potential and permeability of inner and outer membranes, and the release of apoptotic factors in mitochondria.^49^-^52^ By immunofluorescence staining and laser confocal microscopy, we found that JNK localized in mitochondria increased significantly in shikonin treated SW620 cell, which indicated that shikonin could promote JNK to enter mitochondria and regulate mitochondrial-dependent cell apoptosis (Figure 7E).

### Shikonin suppresses tumor growth in SW620 xenograft models

**Figure 8:**
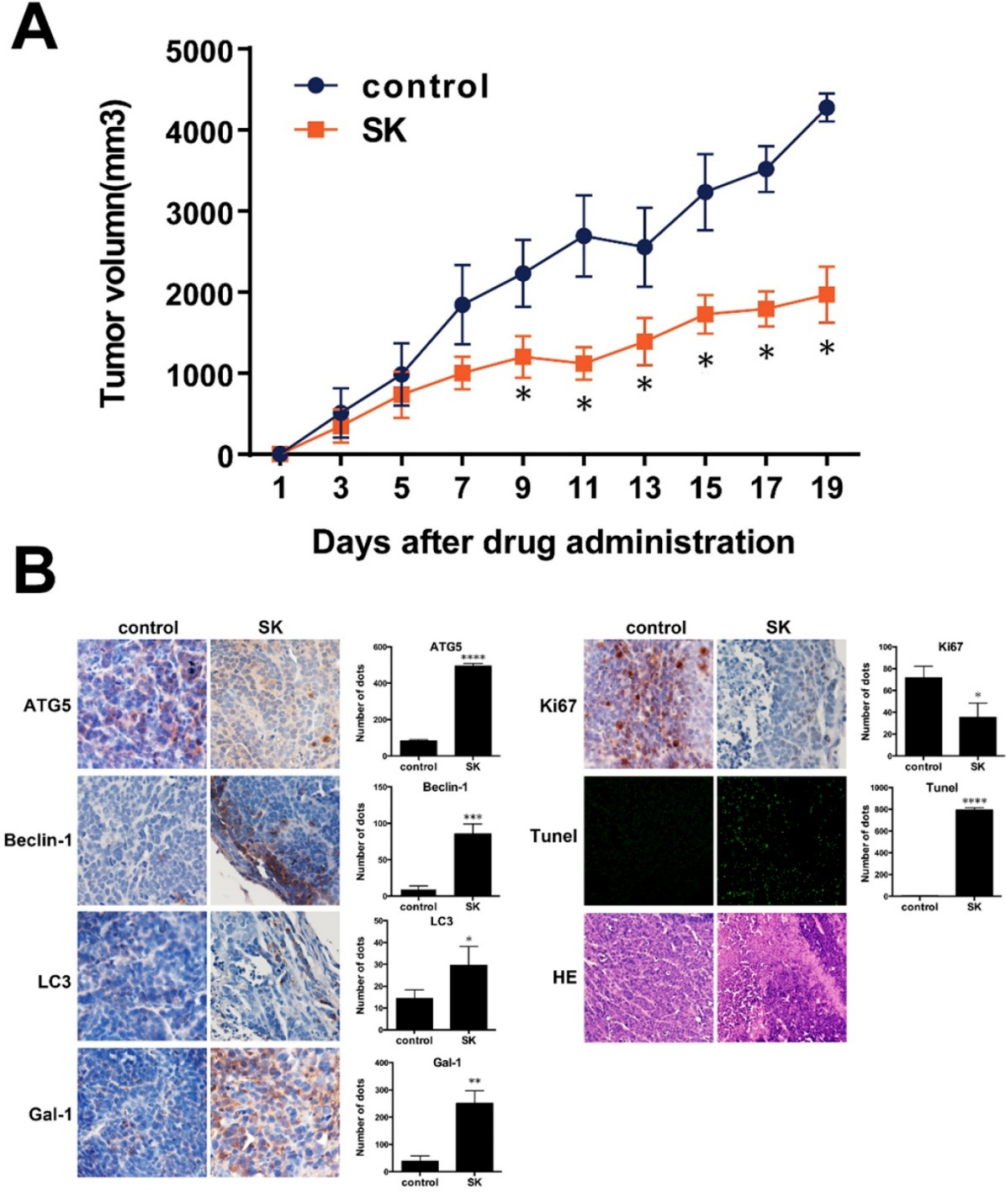
(A) SW620 xenograft tumor volume after treatment with saline (control) or shikonin (SK). Results are mean ± SD; **P < 0.01 and *P < 0.05 vs control (N = 5); (B) Immunohistochemistry to evaluate expression of ATG5, Beclin-1, LC3, galectin-1, Ki67 and Tunel in tumour sections. Quantitation is shown on the right.

According to Fig. 8A, after oral administration of 10 mg/kg shikonin daily for 19 days, the mean tumor volume in shikonin-therapy group was significantly reduced compared to that of the control group with a tumor growth index (TGI) of 52.1% (Figure 8A). Moreover, bodyweight and histological morphology of main organs in shikonin-therapy group were not obviously changed compared to samples from the control group. In the IHC staining of galectin-1, apoptosis- and autophagy-related markers, Ki-67 positive ratio was significantly declined in shikonin-therapy group. While the positive staining of galectin-1, TUNEL, LC3, Beclin-1 and ATG5 were remarkably increased in shikonin-therapy group, these results were consisting with the *in vitro* experiments (Figure 8B).

## Discussion

Shikonin is one of the main active ingredient of traditional chinese medicines Recently, some studies indicate that shikonin has potential anti-tumor effects by inducing programmed cell death, inhibition of cancer cell proliferation, anti-angiogenesis, and shikonin also circumvents cancer drug resistance by inducing necroptotic death.^53^-^57^ In the current study, we found that shikonin induced programmed cell death in colorectal carcinoma cells by activating the expression and dimerization of Galectin-1. We confirmed that shikonin treatment could induce ROS accumulation and apoptosis in human colorectal cancer cells by inhibiting autophagic flux of these tumor cells.

The cell viability and colony formation assays showed that shikonin have good inhibition ability on CRC cell lines (Figure 1A-C). We also observed the apoptotic nuclei condensation induced by shikonin under the fluorescence microscopy directly (Figure 1D). According to the results of flow cytometric, shikonin increased the late stage apoptosis in SW620 and HCT116 cells and the ratios of early apoptosis in HCT116 cell were increased in a dose-dependent manner (Figure 1E). The death receptor apoptosis activates Caspase-8 precursor protein by collecting death handheld protein Fas and adapter protein FADD; the mitochondrial apoptosis activates caspase-8 precursor protein by changing mitochondrial membrane potential and mitochondrial membrane permeability, triggering the release of apoptotic factors such as cytochrome c from mitochondria and forming apoptotic complex with caspase-9 in cytoplasm. These two pathways eventually trigger the activation of caspase-3, enzymatic hydrolysis of apoptotic protease substrates, leading to apoptosis.^58^ In our study, western blot analysis showed that in the two tested CRC cell lines shikonin induced an increase of expression of Fas, FasL, cytochrome c and Bax and led to the cleaved of PARP and caspases (Figure 4A-C). Those evidences suggesting that shikonin can activate the apoptosis of SW620 and HCT116 through both the death receptor pathway and mitochondrial pathway.

Recent studies have shown that autophagy is a potential target for cancer treatment. 59-63 In shikonin treated SW620 and HCT116 cells, we found GFP-LC3 fluorescent puncta was increased when compared with the control group, indicating shikonin induced autophagy in these cells and the conversion of LC3-I to LC3-II was enhanced, and autophagic vacuole was observed by transmission electronic microscopy as well (Figure 2). We also found that shikonin can upregulated the level of LC3 and improved the ratios of LC3-II/LC3-I in SW620 and HCT116 cells. The level of LC3 in cells is usually constant, which may be due to the dynamic balance between the expression and degradation of LC3 in cells. Shikonin-induced elevation of LC3 level may be on account of the dramatic promotion of LC3 expression, which makes the expression rate of LC3 higher than its degradation rate, and makes the level of intracellular LC3 elevated. Rapamycin is a specific mTOR inhibitor that can significantly activate autophagy, and activation of autophagy by rapamycin can promote the autophagy by shikonin in SW620 and HCT116 (Figure 4D). 3-MA can inhibit the formation of autophagosomes by inhibiting class III PI3Ks, while HCQ and bafliomycin can inhibit the degradation of autophagolysosomes.^64^-^66^ In SW620 cell, the inhibition of autophagy by 3-MA can improve the accumulation of LC3 and p62, and inhibited the conversion of LC3-I to LC3-II. When treated with HCQ and bafliomycin, in SW620 cell the shikonin-induced cleavage of LC3 was inhibited and the accumulation of p62 was increased, while in HCT116 cell only HCQ inhibited the shikonin-induced cleavage of LC3 and both of HCQ and bafliomycin improved the accumulation of p62 and LC3 (Figure 4D). Rapamycin enhanced the shikonin-induced cell death might for the reason rapamycin could increase the accumulation of p62 when shikonin inhibits the degradation of autophagolysosomes. HCQ and bafliomycin further inhibit p62 degradation and promote shikonin-induced autophagic death. All these results indicated that shikonin suppressed autophagic flux of colorectal cancer cells and the shikonin-induced autophagy through a canonical pathway.

ROS generation produced by chemotherapy is an important mediator for inducing apoptosis in many kinds of cancer. Here, we demonstrated that ROS accumulation was involved in colorectal carcinoma cell apoptosis. Eliminating ROS via NAC could influence the expression of apoptosis related proteins and autophagy markers, the cleavage of PARP and the level of cytochrome c were decreased and the conversion of LC3 and the expression of p62 were reduced by the treatment of NAC (Figure 5). Moreover, we utilized an iTRAQ-based quantitative proteomic method to identify differentially expressed proteins between SW620 cell with or without shikonin incubation. Galectins and proteins of MAPK pathway both change considerably after shikonin treatment (Figure 3A-C). Further analysis of the mRNA levels of all these proteins also shows large differences between groups (Figure 3D). Interestingly, many reports have supported that galectins have close relationship with proliferation and survival in many kinds of cell types through MAPK pathway.

The growth inhibition effect of Galectin-1 requires interactions with the α5β1 integrin. The inhibition of the Ras-MEK-ERK pathway and the consecutive transcriptional induction of p27 led to anti-proliferative effects. Our data showed that shikonin promotes the dimerization of galectin-1 and the high levels of galectin-1 expression in SW620 and HCT116 cells could make this cell line become more sensitive to shikonin (Figure 6A-F). Also decreasing the amount of dimer galectin-1 in SW620 and HCT116 could reduce the level of cytochrome c and hinder the cleavage of LC3, which resulting in weaken the antitumor effects of shikonin (Figure 6G-I).^67^-^73^ Moreover, we assessed if there was connection between shikonin induced ROS and galectin-1. We finally confirmed that almost all of the dimer galectin-1 disappeared when ROS was cleared by NAC (Figure 6J-K). ROS may be the upstream of galectin-1 in the pathway of shikonin-induced cell death. Besides, we found galectin-1 also participates in the inhibition of migration and invasion by shikonin in SW620 and HCT116. Shikonin could downregulate MMP2 which can cleave or degrade ECM and promot tumor invasion and metastasis, but when decreases galectin-1 though shikonin fail to do it (Figure 6L-M). These results confirmed shikonin could inhibit colorectal cancer cells invasion and Galectin-1 play an important role.

**Figure 9:**
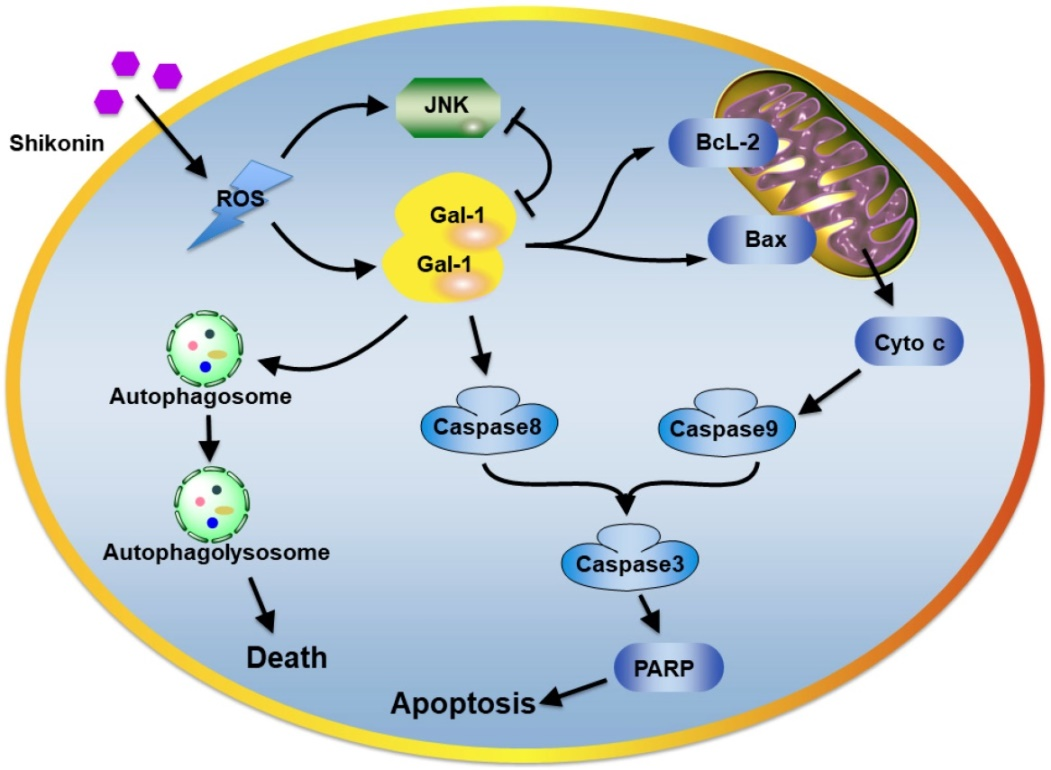
The plausible molecular mechanism of shikonin in colorectal carcinoma cells.

JNK plays a key role in apoptotic pathways by activating specific transcription factors or different phosphorylation events, and activates apoptotic signals by up-regulating apoptotic genes. In SW620 cell, we found shikonin could promote the translocation of JNK to mitochondria which may enhanced the mitochondrial-dependent cell apoptosis (Figure 7E). In our research, we also treated CRC cells with JNK inhibitor SP600125 and found that the shikonin-induced dimerization of galectin-1 increased and the shikonin-induced apoptosis and autophagy of SW620 and HCT116 were influenced. In both the two cell lines, shikonin-induced LC3 conversion was enhanced and the degradation of p62 was promoted, suggesting the inhibition of JNK accelerates the progress of autophagy (Figure 7A). JNK inhibition decreased the release of cytochrome c but the cleavage of caspase-8 was not influenced, which means JNK inhibition may suppress the mitochondrial pathway without retardanting the death receptor pathway. In SW620 cell, cleaved PARP was decrease with the combined treatment of SP600125 and shikonin, but the HCT116 cell show an upregulation of cleaved PARP under the same treatment. These different results may because the inhibition of mitochondrial pathway influenced the total level of apoptosis in SW620 cell, but the activation of death receptor pathway in HCT116 cell can cover the inhibition of mitochondrial pathway (Figure 7B). Furthermore, our research found that the phosphorylation of JNK induced by shikonin could be inhibited in NAC treated CRC cells (Figure 7C). In HCT116 cell, we found the downreguletion of galectin-1 could increase the phosphorylation of JNK while SW620 cell did not show the same results (Figure 9).

In addition, the intracorporal administration of shikonin potently suppressed tumor growth in SW620 xenograft models, which provided evidence that shikonin may be a potential antitumor agent. Further, shikonin suppressed tumor growth and promoted apoptosis and autophagic cell death in tumor tissues, and also upregulated the level of galectin-1 in tumor tissues and could a promising target for shikonin (Figure 8).

In conclusion, our results demonstrated novel target proteins and molecular mechanisms of a naphthoquinone natural product shikonin. The activation of Galectin-1 induced by shikonin potently activated apoptosis in colorectal carcinoma cells and autophagic cell death both *in vitro* and *in vivo*. Our findings suggest shikonin could be chemotherapy for colorectal carcinoma. The molecular basis for targeting galectin-1 as part of programmed cell death regulation of autophagy and apoptosis.

## Supplementary Material

Supplementary figures and tables.
