# Supplementary material for: Shikonin induces colorectal carcinoma cells apoptosis and autophagy by targeting galectin-1/JNK signaling axis
Source: Int J Biol Sci. 2020 Jan 1;16(1):147–61. doi: 10.7150/ijbs.36955 (PMC6930377; doi:10.7150/ijbs.36955)
Supplement: Supplementary file 1 — Supplementary figures and tables. [file ijbsv16p0147s1.pdf]

Shikonin induces colorectal carcinoma cells apoptosis and autophagy by targeting galectin-1/JNK signaling axis

Nan Zhang,[1,†] Fu Peng,[2,†] Yujia Wang,[2] Li Yang,[2] Fengbo Wu,[2] Xiaoyun Wang,[2] Cui Ye,[1] Bo Han[1,*] and Gu He[2,*]

[1] State Key Laboratory of Southwestern Chinese Medicine Resources, School of Pharmacy, Chengdu University of Traditional Chinese Medicine, Chengdu 611137, China

[2] State Key Laboratory of Biotherapy and Cancer Center, West China Hospital, and West China School of Pharmacy, Sichuan University, Chengdu 610041, China

† These authors contributed equally.

* Corresponding authors: Bo Han (hanbo@cdutcm.edu.cn); Gu He (hegu@scu.edu.cn); Tel: +86-28-85503817

Supporting Information

Materials and Methods

Cell extracts and Western blot analysis

After various treatments, cells were harvested by trypsinization and washed with cold PBS. The cells were lysed in RIPA buffer (20 mM Tris–HCl, pH 7.5, 150 mM NaCl, 1 mM Na2EDTA, 1mM EGTA, 1%Triton, 2.5 mM sodium pyrophosphate, 1 mM β-glycerophosphate, 1 mM Na3VO4, 1 μg/ml leupeptin, 1 mM phenylmethylsulfonyl fluo-ride) on ice for 30 min followed by sonication denaturation. Cell lysates were then centrifuged at 13,000 g for 30 min at 4 °C. Collected the supernatant and then protein concentration was determined using a bicinchoninic acid protein assay kit (Thermo, USA). The protein was applied to a 10-15% SDS-polyacrylamide gel, transferred to a nitrocellulose membrane, and then detected by the proper primary and secondary antibodies before visualization by chemiluminescence Kit (Millpore, USA).

RNA extraction and Quantitative real-time PCR

TRIzol® Reagent (Invitrogen, CA, USA) was used to extract total RNA from cells. The protocol of RNA extraction and quantitative real-time PCR were carried out following the manufacturer's protocol. Primer sequences are shown in Supplementary table 1. The relative expression value of GAPDH in each sample was calculated and compared. The experiments were performed in triplicates. All procedures were performed according to the manufacturer's instructions.

Statistical analysis

The statistical analysis was performed using Prism 6.0 software. Comparisons of tumor volume was performed using t test. A P value of <0.05 on a 2-tailed test was considered statistically significant.

Table S1. The differential expressed proteins identified by iTRAQ.

UNIPROT_ACCESSION	gene_symbole	Log2(FC)Sample1	Log2(FC)Sample2
A0A024R035	C9	-2.65745	-2.8039
V9HWA9	C3	-2.39214	-2.61794
P35527	KRT9	-2.39214	-1.3288
D9ZGG2	VTN	-2.20556	-4.1976
B4DR61	SEC61A1	-2.05949	-1.5679
Q5TZZ9	ANXA1	-1.91675	-1.19595
P02751	FN1	-1.7539	-1.83393
Q2M1J6	OXA1L	-1.63442	-2.15264
Q5RKT7	RPS27A	-1.57132	-1.44851
E9PIT3	F2	-1.5679	-1.38195
Q6FHM4	Cox5b	-1.46156	-1.11622
A0A024R254	MAGED1	-1.36848	-1.22527
P31689	DNAJA1	-1.18246	-1.20923
P30048	PRDX3	-1.09031	-1.13073
B2RCP4	IP6K2	-1.08957	-2.00636
Q6FHS4	DNAJB1	-1.08957	-1.95344
P27824	CANX	-1.08957	-1.16942
X5D945	HRAS	-1.05048	-1.04342
Q9P035	HACD3	-1.04961	-1.92687
Q9P0J0	NDUFA13	-1.04961	-1.09793
P51149	RAB7A	-1.0246	-1.07761
Q6NZ44	FTH1	-1.0098	-1.0084
P63241	EIF5A	-1.009849	-1.070702
V9HWG9	GSTO1	-1.009849	-1.979879
O14737	PDCD5	1.023184	1.129481
Q8N122	RPTOR	1.023184	1.129481
Q9Y3U8	RPL36	1.036468	1.06302
V9HW72	STIP1	1.036468	1.089566
P31947	SFN	1.036468	1.265797
Q02790	FKBP4	1.036468	1.488206
P30044	PRDX5	1.044782	1.264392
Q76LA1	CSTB	1.050399	1.594549
P61960	UFM1	1.06302	1.408494
A0A024R324	RHOA	1.06302	1.820404
P13010	XRCC5	1.076285	1.235727
Q14444	CAPRIN1	1.076285	1.820404
E7EUC7	UGP2	1.089566	1.793854
P62826	RAN	1.10286	1.116165

Q9Y266	NUDC	1.10286	1.581255
P61244	MAX	1.10286	1.581255
Q05639	EEF1A2	1.129481	1.170702
P49903	SEPHS1	1.156008	1.116165
V9HWH1	SERPINB1	1.169348	1.116165
P34932	HSPA4	1.169348	1.528071
V9HWD6	YWHAB	1.169348	1.621102
Q6PKG0	LARP1	1.171337	1.332164
V9HWH7	ATIC	1.182629	1.023184
F8WCF6	ARPC4-TTLL3	1.182629	1.275603
P0CW22	RPS17	1.182629	1.408494
O14907	TAX1BP3	1.195914	1.036468
A0MZ66	SHTN1	1.195914	1.182629
A4D2P1	RAC1	1.196606	1.183313
Q53SS8	PCBP1	1.209204	1.156008
Q9H074	PAIP1	1.222495	2.165912
D0PNI1	YWHAZ	1.222495	3.388369
K7EQ55	DAZAP1	1.235727	1.14274
V9HW96	CCT2	1.235727	1.421802
B2ZZ89	SPTBN1	1.235727	1.541366
P04637	TP53	1.235727	1.541366
Q5SRT3	CLIC1	1.249021	1.700839
J3KT51	HN1	1.262313	1.262313
Q06830	PRDX1	1.275603	1.06302
P53618	COPB1	1.275603	1.408494
V9HW37	CCT5	1.275603	1.860287
P25786	PSMA1	1.28889	1.421802
P17931	LGALS3	1.28889	1.169348
F4ZW62	ILF2	1.302173	1.14274
Q6NUR7	EZR	1.302173	1.28889
Q5U077	LDHB	1.355355	1.767273
Q9ULC4	MCTS1	1.368657	1.089566
P23526	AHCY	1.368657	1.315508
P50991	CCT4	1.368657	1.581255
P13797	PLS3	1.381948	1.979879
I4AY87	MIF	1.381948	2.21906
P63208	SKP1	1.408494	1.009849
O00154	ACOT7	1.408494	1.395227
V9HWC0	MSN	1.435095	1.156008
V9HW98	YWHAE	1.435095	1.421802
Q9H1Y0	ATG5	1.435095	1.156008
Q9UNZ2	NSFL1C	1.474929	1.926721
P07737	PFN1	1.501516	1.581255
Q6IAT1	GDI2	1.501516	1.873577

Q9BZZ5	API5	1.514804	2.099464
Q53YD7	EEF1G	1.528071	1.116165
V9HWD9	TKT	1.528071	1.262313
Q06323	PSME1	1.541366	1.953302
V9HWI0	AKR1A1	1.554638	1.554638
P01106	MYC	1.554638	1.554638
Q0VAS5	Hist1h4h	1.567935	1.421802
V9HWF4	PGK1	1.581255	2.033018
Q15691	MAPRE1	1.594549	1.06302
Q6FGD7	TBCA	1.594549	1.421802
Q9H0U4	RAB1B	1.607816	1.292311
Q6PKH8	ANP32A	1.621102	2.710702
P52209	PGD	1.634407	1.461633
V9HWB8	PKM	1.660974	1.873577
V9HVZ4	GAPDH	1.674235	1.514804
H0YIV4	NAP1L1	1.674235	1.793854
V9HWE9	GSTP1	1.674235	2.139306
Q16658	FSCN1	1.687553	1.10286
H9ZYJ2	TXN	1.687553	1.116165
P60174	TPI1	1.687553	1.966578
E9PK25	CFL1	1.740669	1.873577
P40925	MDH1	1.75399	1.860287
P49773	HINT1	1.767273	1.993167
Q13501	SQSTM1	1.780562	1.116165
P12814	ACTN1	1.793854	2.205768
V9HWF5	PPIA	1.8337	1.674235
J3KN48	ATP6V0E1	1.846995	2.644249
V9HWC7	PRDX6	1.860287	1.886862
O75506	HSBP1	1.873577	1.169348
D3DPU2	CAP1	1.886862	1.581255
P04049	RAF1	1.900142	2.976455
P00338	LDHA	1.900142	1.474929
P62269	RPS18	1.900142	2.258911
V9HWK2	VCL	1.913416	1.767273
Q14457	BECN1	1.940016	1.14274
Q32Q12	NME1-NME2	1.953302	1.461633
P37837	TALDO1	1.993167	2.298776
D3DSW3	PROSC	2.099464	1.528071
P09382	LGALS1	2.137842	2.156008
D9IAI1	PEBP1	2.165912	2.192478
V9HW44	PAFAH1B2	2.910003	1.488206

Figure S1. The colony formation of SW620 and HCT116 cells after shikonin incubation;.

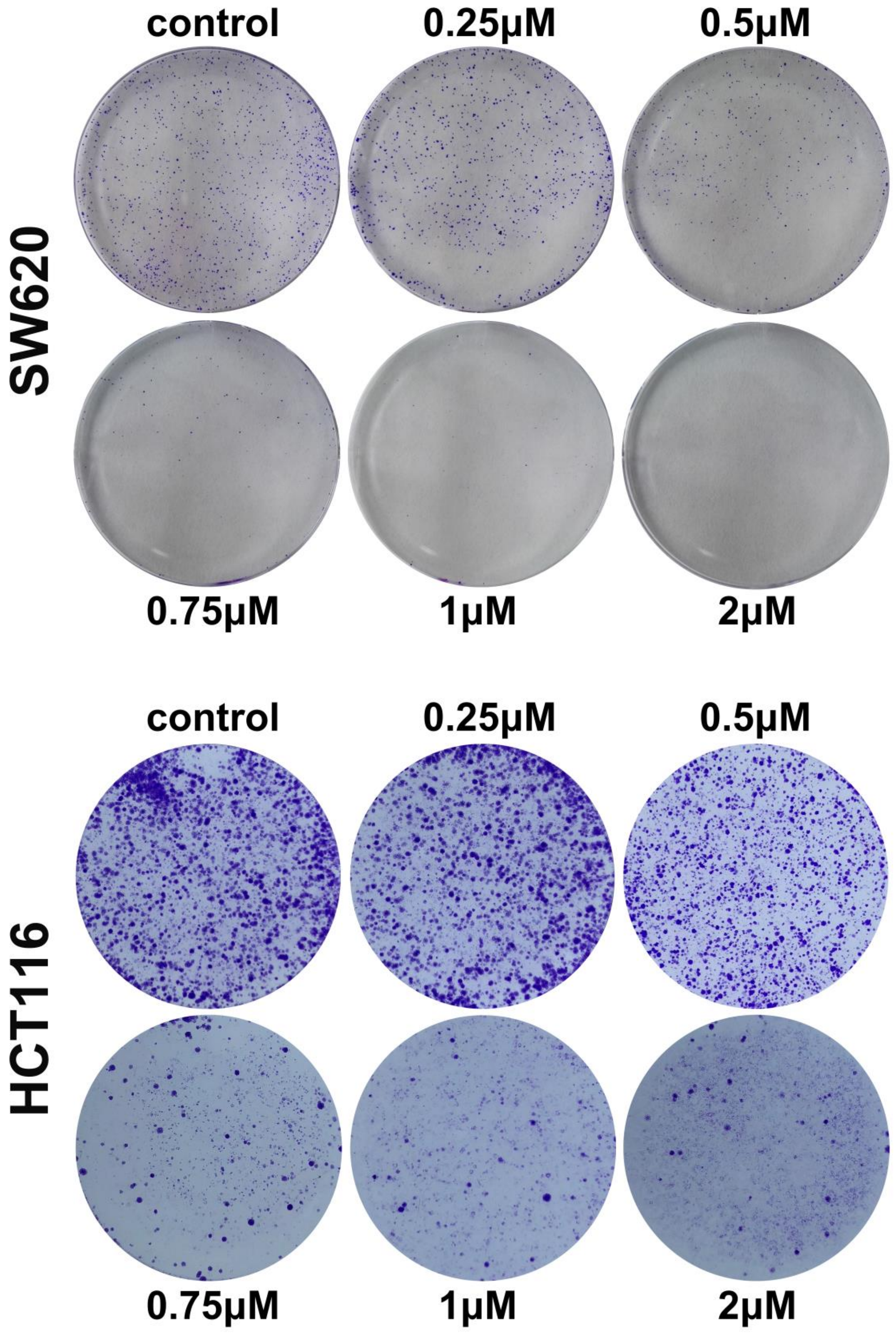

Figure S2. The ROS accumulation induced by Shikonin in HCT116 and SW620 cells.

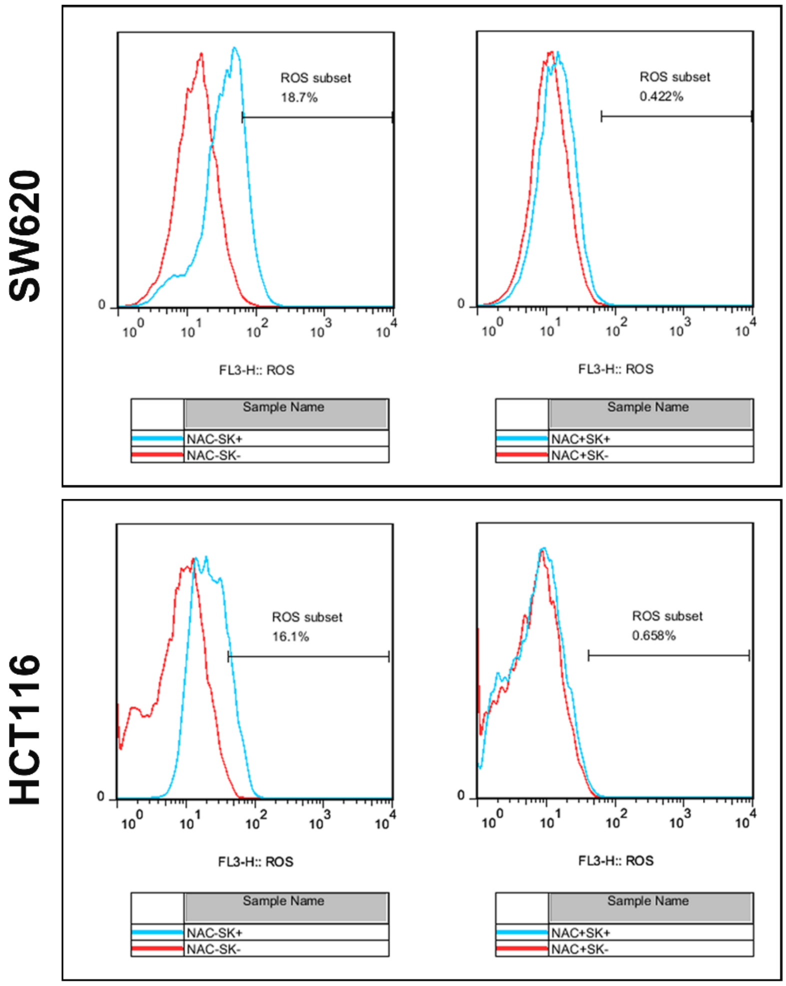

Table S2. The sequences of primers for qRT-PCR.

Gene Name	Sequence of upstream primer	Sequence of downstream primer	Amplified fragment size (bp)
GAPDH	TGACTTCAACAGCGACACCCA	CACCCTGTTGCTGTAGCCAAA	121
RPTOR	AACCGAAACCCACCCGAAC	GGAAGATGCCGACAGACAAGG	145
HRAS	CAGATCAAACGGGTGAAGGAC	GCCTGCCGAGATTCCACAG	95

CDKN1B	TAATTGGGGCTCCGGCTAACT	TGCAGGTCGCTTCCTTATTCC	116
LGALS1	CTGGAAGTGTTGCAGAGGTGT	CTGGCTGATTTCAGTCAAAGG	174
MAPK14	CTGTTGGACGTTTTTACACCTGC	AGACCTCGGAGAATTTGGTAGA	158
RAC1	ATGTCCGTGCAAAGTGGTATC	CTCGGATCGCTTCGTCAAACA	249
MAPK3	ATGTCATCGGCATCCGAGAC	GGATCTGGTAGAGGAAGTAGCA	156
RAB1A	TTTGGAAACCAGTGCTAAGAATGC	GCTGTGAGAAAAGGATGGAGGC	196
CDK6	CCTTAGCACAGCACCAC	GGGATTTCTCAGCCAGT	167
MAX	CGGGCTCATCATAATGCACTG	GGACTGAGTCCCGCAAACTG	79
TP53	GAGGTTGGCTCTGACTGTACC	TCCGTCCCAGTAGATTACCAC	133
MAPK1	TTACGACCCGAGTGACGA	CTGTATCCTGGCTGGAATCT	129
ATG5	AGAAGCTGTTTCGTCCTGTGG	AGGTGTTTCCAACATTGGCTC	152
BECN1	GCTGCCGTTATACTGTTCTG	TGCCTCCTGTGTCTTCAATC	182
CDK2	CTGGACACTGAGACTGAGG	GAGGACCCGATGAGAATGG	264
LGALS3	GAGCCTACCCTGCCACTG	CGACTGTCTTTCTTCCCTTCC	269
NFKB1	AGGATTTCGTTTCCGTTATGT	CCTGAGGGTAAGACTTCTTGTTC	92
CDKN1A	GGGACAGCAGAGGAAGACC	GACTAAGGCAGAAGATGTAGAGC	260
RAB7A	GTCGGGAAGACATCACTCA	CTAGCCTGTCATCCACCAT	115
PRDX1	CATTCCTTTGGTATCAGACCCG	CCCTGAACGAGATGCCTTCAT	83
CDK4	AGTGGTGGAACAGTCAAG	AGCCCAATCAGGTCAAAG	248
LGALS7	TCCATGTAAACCTGCTGTGCG	CCTTGCTGTTGAAGACCACCT	99
MYC	TGTCCGTCCAAGCAGAGG	CGCACAAGAGTTCCGTAGC	107

MAPK8	GTGGATTTATGGTCTGTGGG	AAGTCCTTACTGTTGGTTGC	160
RHOA	AAGCATTTCTGTCCCAACG	TCACAAGACAAGGCACCC	273
SQSTM1	GACTACGACTTGTGTAGCGTC	AGTGTCCGTGTTTCACCTTCC	139
MXD1	AGCACGGACTATCTCACAGGT	GGTGGGACACTGAAGTTTACG	275
MAP3K5	CTGCATTTTGGGAAACTCGACT	AAGGTGGTAAAACAAGGACGG	120
RAF1	CCAGGAGACCAAGTTTCAGATG	CAGAGGACAGAGCCAGTAGG	228
